# To Contributors

**Published:** 1867-04

**Authors:** 


To Contributors.—Interesting communications have been received which will appear in our next number. Correspondents and contributors will be patient with us; our pages have recently been provided for a long time in advance of publication.

☞ We have received the first numbers of the *American Naturalist*, a journal devoted to the interest of Natural Science, to which we desire to call the especial notice of those of our readers who are interested in the advances so rapidly being made in this department.

